# Plant Soft Rot Development and Regulation from the Viewpoint of Transcriptomic Profiling

**DOI:** 10.3390/plants9091176

**Published:** 2020-09-10

**Authors:** Ivan Tsers, Vladimir Gorshkov, Natalia Gogoleva, Olga Parfirova, Olga Petrova, Yuri Gogolev

**Affiliations:** 1 Laboratory of plant infectious diseases, Federal Research Center Kazan Scientific Center of Russian Academy of Sciences, 420111 Kazan, Russia; i.Tsers@knc.ru; 2 Kazan Institute of Biochemistry and Biophysics, Federal Research Center Kazan Scientific Center of Russian Academy of Sciences, 420111 Kazan, Russia; gogoleva@kibb.knc.ru (N.G.); olga.parfirova@kibb.knc.ru (O.P.); olga.petrova@kibb.knc.ru (O.P.); gogolev@kibb.knc.ru (Y.G.)

**Keywords:** plant-microbe interactions, phytoimmunity, induced susceptibility, *Pectobacterium atrosepticum*, RNA-Seq, transcription factors

## Abstract

Soft rot caused by *Pectobacterium* species is a devastating plant disease poorly characterized in terms of host plant responses. In this study, changes in the transcriptome of tobacco plants after infection with *Pectobacterium atrosepticum* (*Pba*) were analyzed using RNA-Seq. To draw a comprehensive and nontrivially itemized picture of physiological events in *Pba*-infected plants and to reveal novel potential molecular “players” in plant–*Pba* interactions, an original functional gene classification was performed. The classifications present in various databases were merged, enriched by “missed” genes, and divided into subcategories. Particular changes in plant cell wall-related processes, perturbations in hormonal and other regulatory systems, and alterations in primary, secondary, and redox metabolism were elucidated in terms of gene expression. Special attention was paid to the prediction of transcription factors (TFs) involved in the disease’s development. Herewith, gene expression was analyzed within the predicted TF regulons assembled at the whole-genome level based on the presence of particular cis-regulatory elements (CREs) in gene promoters. Several TFs, whose regulons were enriched by differentially expressed genes, were considered to be potential master regulators of *Pba*-induced plant responses. Differential regulation of genes belonging to a particular multigene family and encoding cognate proteins was explained by the presence/absence of the particular CRE in gene promoters.

## 1. Introduction

Soft rot *Pectobacteriaceae* (SRP), including *Pectobacterium atrosepticum* (*Pba*), cause devastating plant diseases [1]. These bacteria produce plant cell wall-degrading enzymes (PCWDEs) leading to extensive plant tissue maceration [2]. SRP have long been considered to be necrotrophic pathogens that attack their hosts by brute force and not by stealth through manipulation of host plant responses as bio- and hemibiotrophic pathogens do. However, in fact, all plant pathogenic bacteria (except for biotrophic agrobacteria) are hemibiotrophs [3] and, therefore, possess specific virulence factors that enable them to modulate host plant responses. *Pectobacterium* species, in addition to PCWDEs, produce virulence factors typical of the stealth mode of action (e.g., type III and VI secretion systems, coronafacic acid) [4,5,6,7,8,9]. This indicates that the colonization of plants by pectobacteria is coupled with significant alterations in host plant physiology. The general picture of these alterations is not clear and no genetic determinants of plant resistance/susceptibility have been revealed to date.

*Pba*-induced plant responses related to plant cell wall modification have been described in our previous studies by the example of tobacco plants that display typical soft rot symptoms after inoculation with *Pba* [10,11]. These responses provide a host-mediated reconstruction of plant cell walls associated with the release of one of the pectic compounds—rhamnogalacturonan I (RG-I)—into the lumen of xylem vessels. When inside the vessel, RG-I serves as a matrix for bacterial cells contributing to the colonization of the vascular system. However, plant gene products that could implement this reaction remain unknown.

To draw a comprehensive physiological portrait of an organism, the RNA-Seq approach is often applied [12,13]. One of the crucial bottlenecks of RNA-Seq analysis is poor algorithms for data interpretation in terms of general physiology. The analysis is usually restricted to automatic sorting of differentially expressed genes (DEGs) employing popular databases, such as Gene Ontology (GO) or Kyoto Encyclopedia of Genes and Genomes (KEGG). Such an approach usually yields quite a superficial description of the results that covers only “the tip of the iceberg”.

Our study was aimed at gaining a better understanding of the physiological events in *Pba*-infected tobacco plants based on RNA-Seq data. The original gene sorting based on automatic classification was supplemented by detailed manual sorting in order to identify genes whose products might determine previously described physiological reactions in *Pba*-infected plants and to reveal novel molecular aspects of plant–*Pba* interactions. Special attention was paid to the prediction of transcription factors (TFs) involved in the disease’s development. The original pipeline that expands the capabilities of transcriptome analysis was developed to monitor gene expression within the predicted regulons of different TFs. This allowed us to reveal candidate master regulators of the infection and to explain possible reasons for the differential regulation of genes belonging to the particular multigene families within which both up- and downregulated genes were well-represented.

## 2. Results and Discussion

**Figure 1:**
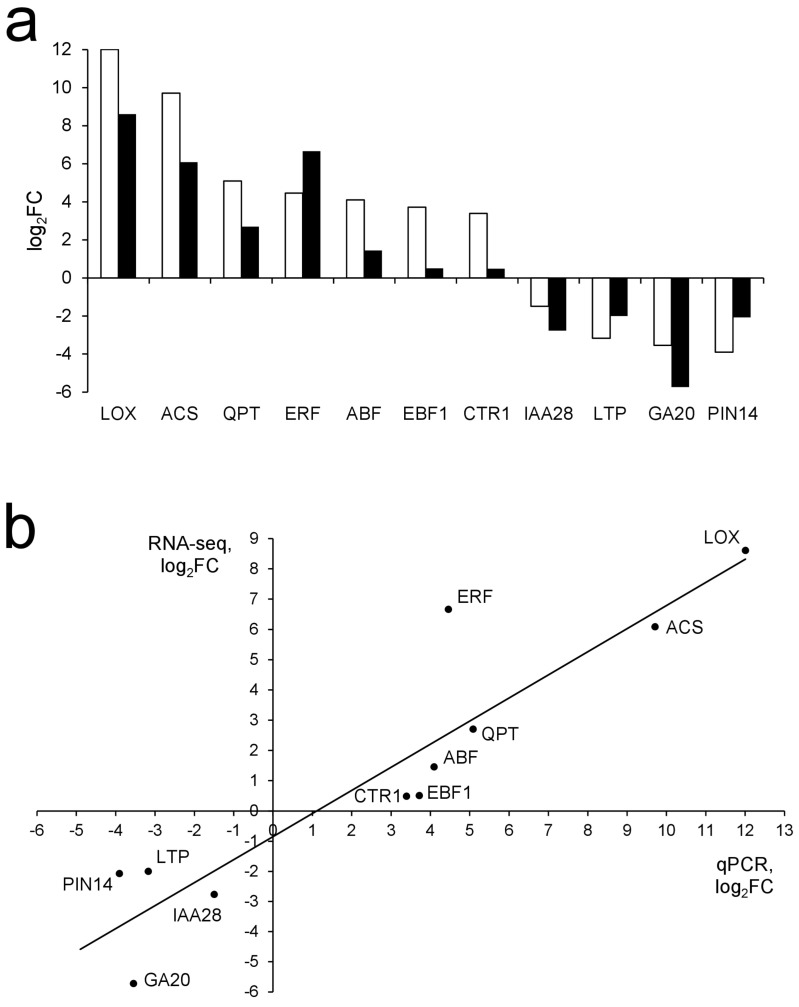
Verification of RNA-Seq data by qPCR. (**a**) Log_2_Fold Changes measured by qPCR (white bars) and RNA-Seq (black bars); (**b**) Correlation of log_2_FC values calculated based on RNA-seq (X-Axis) and qPCR (Y-Axis) data. Pearson’s *r* = 0.92 and is significant at *p* < 0.001.

The results of RNA-Seq read processing, mapping, and quantification in each sample are given in Supplementary Table S1. In the samples of *Pba*-infected tobacco plants, compared with uninfected ones, 8636 differentially expressed genes (DEGs) were revealed: 3797 were upregulated and 4839 were downregulated. The hierarchical clustering of sample-to-sample Euclidean distances showed that the sample replicates were consistent with each other within their respective groups (Supplementary Figure S1). The RNA-Seq results correlated with the qPCR data (Pearson’s *r* = 0.92) (Figure 1).

DEGs were split into different categories and subcategories (Supplementary Table S2) according to their classification (MapMan, KEGG, and CAZy) and annotation (SwissProt) in different databases. The gene categories and subcategories assembled in our study based on merging the information from different sources are described below.

### 2.1. Categories of DEGs

#### 2.1.1. Cell Wall

We attributed 620 DEGs to the “Cell wall” category, which was additionally subdivided into different subcategories (Supplementary Table S2). The majority of genes encoding the catalytic subunits of *cellulose* synthase A (CesA) were downregulated and none of them were upregulated. Downregulation was especially pronounced for the secondary cell-wall-related CesAs CesA4 (LOC107817758), CesA7 (LOC107806628), and CesA8 (LOC107830312). Genes for COBRA-like proteins (considered to be involved in cellulose synthesis or deposition, [14]) were also downregulated. Therefore, the synthesis of cellulose seemed to be repressed during the infection.

Almost all DEGs whose products are involved in the synthesis of *cross-linking glycans* (CLGs) (xyloglucan, xylan, glucomannan) were downregulated. The exception was a minor group of genes with low expression levels (TPM values) encoding glycosyltransferases from the CSL and exostosin families. DEGs encoding various CLG hydrolases were also mostly downregulated except for some xyloglucan endotransglycosylases/hydrolases (XTHs) and some endoglucanases. XTHs are able to cleave and then rejoin xyloglucan chains providing cell wall loosening during cell growth and fruit ripening [15]. Some members of the XTH family have been shown to act in plant–pathogen interactions, but their specific role is unknown. The expression of the XTH1 gene was reported to be induced in susceptible plants but repressed in resistant plants [16]. In our study, genes for XTH23 (LOC107794227 and LOC107786882), XTH27 (LOC107815234), XTH33 (LOC107789579), and XTH30 (LOC107763397 and LOC107762311) as well as one of the XTH2 isoforms (LOC107781642) were upregulated during the infection. In addition, some endoglucanases that may be “directed” towards either plant CLGs or glucans of pathogen origin were upregulated. Thus, the infection was likely associated with the repression of CLG metabolism (both synthesis and degradation), except that some XTH and endoglucanase genes were upregulated.

The most abundant group of DEGs in the “Cell wall” category was related to the *pectic polysaccharides* polygalacturonan (PG) and rhamnogalacturonan (RG) (RG-I and RG-II). The biosynthetic genes for both PG- and RG-related glycosyltransferases were mostly downregulated during the infection. As for the PG- and RG-degradation-related genes, both up- and downregulated ones were revealed. DEGs for pectate lyases were downregulated, while DEGs encoding polygalacturonases were represented by both induced and repressed ones. Some DEGs encoding RG-degrading enzymes (RG lyases (LOC107816998 and LOC107760147) and beta-galactosidases (LOC107812610, LOC107779266, LOC107778191, and LOC107762980)) were strongly upregulated up to log_2_FC ≈ 13. Most of the genes encoding pectin methyl/acetyl esterases were downregulated, but some of them were induced.

Thus, the gene expression profile showed that the synthesis of pectic compounds was evidently repressed during the infection. As for pectin degradation/modification, in general, it was also likely repressed. However, some “sides” of pectin modification were enhanced, namely the modification of RG by RG lyases and beta-galactosidases as well as PG by polygalacturonases. Several pectin-degrading/modifying enzymes of the host plants were previously shown to play a role in the induced plant susceptibility to fungal pathogens exacerbating disease development [17,18,19]. Therefore, the revealed plant RG lyases, beta-galactosidases, and polygalacturonases induced during *Pba*-caused infection probably serve as susceptibility factors that contribute to the loosening of plant cell walls that promotes pathogen propagation.

DEGs related to biosynthesis and deposition of *callose* were all downregulated. These genes included several ones for callose synthases (CALSs) and PLASMODESMATA CALLOSE-BINDING PROTEINs (PDCBs). The latter were shown to increase callose accumulation and decrease molecular diffusion via plasmodesmata [20]. Thus, although callose deposition is considered to be a hallmark of biotic stress, we did not find any evidence of increased callose biosynthesis during *Pba*-caused infection that is consistent with the depression of callose deposition during *Pectobacterium-carotovorum*-caused infection [21].

Many DEGs related to *lignification* of the plant cell wall were induced during the infection. Genes encoding the enzymes that catalyze the synthesis of monolignols were mostly upregulated. In addition, genes encoding BGLU beta-glucosidases involved in the deglycosylation of inactive glucosylated monolignols were upregulated. In turn, it was unlikely that glucosylation of monolignols took place since genes encoding the UDP glucosyltransferases UGT84A2, UGT84A3, UGT72E2, and UGT72E3 reported to be active toward the monolignols [22,23] were not found among the expressed genes in our experiments. DEGs related to the polymerization of monolignols were also mostly induced. Almost all of the DEGs for lignin peroxidases demonstrated pronounced upregulation (log_2_FC from 3 to 10). Genes encoding laccases were split into two groups: a repressed one (10 genes encoding LAC4, LAC6 (LOC107790401 and LOC107760106), LAC11 (LOC107789055 and LOC107792419), and LAC17 (LOC107769975, LOC107802478, LOC107808047, and LOC107791207)) and an induced one (LAC14 (LOC107830713 and LOC107784916) and LAC7 (LOC107792673 and LOC107818683)). Therefore, according to the gene expression profile, lignin monomers were likely both synthesized de novo and mobilized from their glucosylated derivatives during the infection. The polymerization of monolignols could have occurred due to the induction of genes for laccases of several families and strong transcriptional upregulation of lignin peroxidase genes.

Many DEGs were related to *plant cell wall proteins*: arabinogalactan proteins, trichome birefringence-like proteins, chitinase-like proteins, extensins, and expansins. The exact functions of arabinogalactan proteins (AGPs), the abundant O-glycosylated cell wall proteins, remain unclear. However, it is known that the profile of AGPs is altered depending on the physiological state/reaction with upregulation of some AGP groups and downregulation of others [24]. Interestingly, in our study, almost all of the AGP DEGs (37 of 39) were downregulated during the infection. This means that the *Pba*-caused infection is coupled with a systemic decrease in AGP level, except for the two upregulated AGP4-like proteins encoded by LOC107790227 and LOC107760090. Genes for trichome birefringence-like (TBL) proteins were also systemically downregulated (47 of 50 genes). The exact functions of TBL proteins are also unknown, but they were suggested to participate in cellulose crystallization and xyloglucan modification [25,26]. Therefore, the downregulation of TBL genes during the infection is consistent with the repression of cellulose- and CLG-related genes.

Two-thirds of the genes encoding chitinase-like proteins were upregulated. Although chitinases are well-known resistance factors, their action is unlikely to be restricted to polymers of pathogens since chitinase gene upregulation takes place during normal plant development [27,28]. Thus, the upregulation of chitinase genes may be related to both direct counterattacks against *Pba* and *Pba*-induced modification of the cell wall.

DEGs encoding extensins that strengthen plant cell walls by forming a rigid network were distributed approximately equally between up- and downregulated genes. This means that the profile of extensins in the course of infection may be altered, but whether these changes make a contribution to cell wall rigidity remains to be determined. Expansin (EXP) genes were also represented by up- and downregulated ones. EXPs provide the breakdown of hydrogen bonds between cellulose microfibrils and CLG allowing for the polymer creep and cell wall loosening that is crucial, in particular, to cell growth and fruit ripening [29]. EXPs are divided into four families: EXPA, EXPB, expansin-like A (EXLA), and expansin-like B (EXLB). In our experiments, all DEGs encoding EXPAs (LOC107773912, LOC107802488, LOC107787480, LOC107818570, and LOC107812998) were downregulated, while all DEGs encoding EXLBs (LOC107831803, LOC107823695, LOC107779922, LOC107821371, LOC107794363, and LOC107781933) were upregulated. This means that *Pba* is likely to exploit host EXLBs for plant cell wall loosening. EXPs (namely EXPAs and EXLA) were previously shown to act as susceptibility factors in *Xanthomonas* sp. and *Botrytis cinerea* infections [30,31,32]. Our results show that EXLBs may also act as susceptibility factors assisting *Pba* with plant colonization.

The plant cell wall is a battleground for plant–pathogen interactions [33]. On the one hand, a plant strengthens its cell walls to restrict pathogen propagation. On the other hand, a pathogen may activate plant cell wall loosening not only by its own enzymes/proteins but also by manipulating the host’s ones in the framework of plant susceptible response [34]. Our analysis revealed both sides of plant cell wall modification. The “defense side” is evidently related to the induction of lignification-related genes and chitinase-encoding genes as well as the repression of genes for pectinesterases that make pectic compounds more available to microbial enzymes. Alterations in the extensin profile may presumably also contribute to the resistance by increasing cell wall rigidity. In turn, several observed changes in gene expression likely reflect the cell-wall-related susceptible plant response, namely the activation of genes encoding the enzymes and proteins involved in cell wall loosening (XTHs, EXPs) and the repression of genes related to the synthesis of cellulose, CLG, and callose.

**Figure 2:**
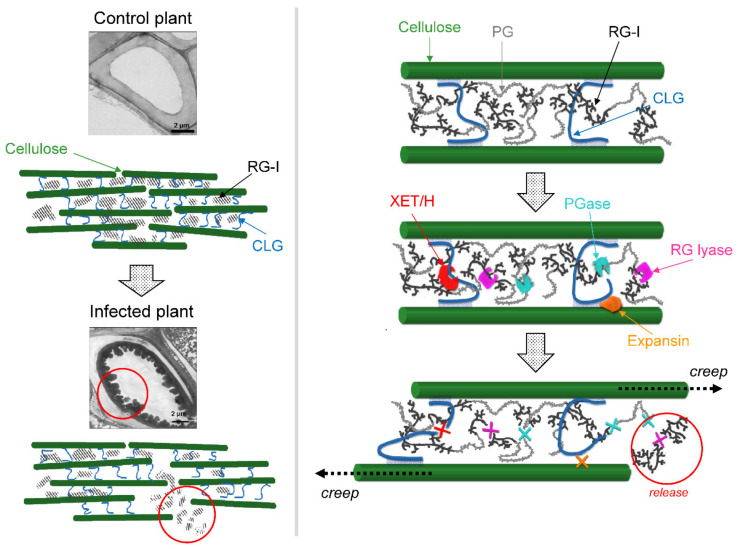
Hypothetical scheme of plant-mediated plant cell wall loosening that leads to the release of rhamnogalacturonan I (RG-I) fragments into the vessel lumen as a result of plant responses to *Pectobacterium atrosepticum*. **Left-hand side, top**: photo of the vessel of the control plant and scheme of the intact cell wall structure; **bottom**: photo of the vessel of the infected plant showing the release of RG-I (red circle) and scheme for plant cell wall modification associated with the release of RG-I (red circle). **Right-hand side**: scheme of events related to the release of RG-I at the scale of one pair of cellulose microfibrils. Expansins and xyloglucan endo-transglycosylases/hydrolases (XTHs) acting towards cross-linking glycans (CLGs) provide polymer creep and cell wall loosening, while polygalacturonases (PGases) hydrolyzing polygalacturonan (PG) lead to the release of RG-I-rich domains that are further cleaved by RG lyases and galactosidases. Crosses in the bottom panel point to the bonds disrupted by the enzymes or expansins.

Previously, we showed that colonization of the primary xylem vessels by *Pba* associated with the formation of bacterial emboli (biofilm-like “multicellular” structures) is coupled with plant cell wall modification expressed in the release of RG from the plant cell wall into the vessel lumen [10,11]. Within the vessel, RG serves as an extracellular matrix for bacterial emboli assemblage. The release of RG could not be explained by the action of bacterial enzymes, and, therefore, was attributed to the host plant ones [11,35]. The performed transcriptome profiling distinctly points to the mechanism of RG release. RG does not emerge in a vessel lumen due to its de novo synthesis; RG biosynthetic genes are repressed (this study), and the increase in RG content in a buffer-extractable fraction (polysaccharides unbound to the cell wall) is coupled with its decrease in an ammonium-oxalate-extractable fraction (ion-bound pectins) [11]. Therefore, the pre-synthesized RG is released from the plant cell wall. Such a release may be provided by the action of a consortium of gene products, which expression was increased during the infection. The upregulation of XTHs and EXPs may lead to polymer creep that provides the enzymes with access to their substrates and promotes the release of polysaccharide fragments (Figure 2). Polygalacturonases may split RG-rich and PG-rich domains of complex pectic molecules. In turn, RG lyases and beta-galactosidases acting towards the backbone and galactose side chains of RG, respectively, may reduce its molecular weight and promote RG release through the cell wall pores. Thus, our previous data on *Pba*-induced plant cell wall modification and release of RG [11] serve as evidence for the correlation of physiological events and transcriptome alterations in the infected plants. Moreover, these RNA-Seq data deepen our understanding of a previously observed plant cell wall modification by predicting particular gene products involved in it.

#### 2.1.2. Phytohormones

A large group of *ethylene* biosynthetic genes encoding 1-aminocyclopropane-1-carboxylate oxidases (ACOs) and 1-aminocyclopropane-1-carboxylate synthases (ACSs) were strongly upregulated. Genes for ethylene receptors were mostly non-DEGs except for three upregulated ones (LOC107790374, LOC107831604, and LOC107825743). One upregulated gene for the primary ethylene-responsive TF EIL3 (LOC107799778) and plenty of DEGs (mostly upregulated ones) encoding the downstream ethylene-responsive TFs (ERFs) were revealed.

Many DEGs related to the lipoxygenase pathway yielding *jasmonic acid* (JA) and other oxylipins were upregulated with log_2_FC values of 10 to 15 for some of them. Nine genes encoding lipoxygenases that catalyze the formation of fatty acid hydroperoxides—the initial products of the lipoxygenase pathway—were upregulated. Fatty acid hydroperoxides are further converted by several groups of enzymes: allene oxide synthases (AOSs), divinyl ether synthases (DESs), hydroperoxide lyases (HPLs), and peroxygenases [36]. Genes encoding AOSs and other enzymes of the JA-related branch of the lipoxygenase pathway (allene oxide cyclases, 12-oxophytodienoic acid reductases) were upregulated as well as the genes for DESs. DESs catalyze the formation of colneleic and colnelenic acids (divinyl ethers) that were demonstrated to inhibit the growth of some phytopathogenic fungi and bacteria but not that of *Pectobacterium carotovorum* [37]. Therefore, the apparent increase in divinyl ethers due to the dramatic upregulation (log_2_FC up to 15) of several DES genes (LOC107808154, LOC107799697, LOC107766501, and LOC107825278) is unlikely to have a direct antimicrobial effect against *Pba*. It is unlikely that the HPL- and peroxygenase-related branches of the lipoxygenase pathway were induced during the infection. No upregulated genes for HPLs that catalyze the first step of traumatin synthesis were revealed. All of the DEGs encoding peroxygenases (LOC107765104, LOC107791606, LOC107780594, and LOC107808920) that catalyze the formation of epoxy fatty acids including epoxyalcohols that were shown to possess antimicrobial activity [37] were downregulated.

Thirteen upregulated DEGs encoded jasmonate ZIM-domain proteins (JAZ), also known as TIFY proteins—JA-induced co-receptors of JA [38]. Four of the five DEGs encoding the JA-dependent TF MYC2 were upregulated (LOC107767049, LOC107820916, LOC107817883, and LOC107790053). Thus, transcriptome profiling explicitly indicates that the ethylene- and JA-mediated hormonal pathways known to have synergistic action are highly induced during *Pba*-caused infection. Genes related to the lipoxygenase pathway were regulated differentially: genes for JA- and DE-related branches were induced, while genes for traumatin- and epoxyalcohol-related ones were not. To correlate the induction of lipoxygenase-pathway-related genes with the activity of corresponding enzymes, lipoxygenase and divinyl ether synthase activities were measured in control and *Pba*-infected plants. Lipoxygenase and divinyl ether synthase activities were 28-times and 24-times, respectively, higher in the infected plants, confirming the results of the transcriptome profiling.

Although many aspects of *Pba*-caused disease have been described by the example of tobacco plants, the potato plants are considered to be the main host of *Pba*. Therefore, we analyzed if the induction ethylene- and lipoxygenase-pathway-related genes took place in *Pba*-infected potato plants. We showed that these genes were also induced in *Pba*-infected potato plants (Supplementary file 1). Thus, the induction of the lipoxygenase pathway, as well as ethylene-regulated responses, is a typical hallmark of *Pba*-caused disease. This is supported by the fact that the inability of *Pba* to produce coranafacic acid (a functional analog of JA) leads to a reduction in the pathogen’s virulence [35].

As for *salicylic acid* (SA) that can be synthesized via cinnamic acid and via isochorismate, only genes related to the synthesis of its distant precursors were upregulated. Among these genes were those encoding phenylalanine ammonia lyases (LOC107831213, LOC107792668, and LOC107786762) and chorismate mutases (LOC107805472 and LOC107800931) that were involved in other metabolic pathways induced in our experiments (phenylpropanoid and prephenate pathways). Therefore, it was unlikely that the induction of these genes contributed to the SA synthesis. In turn, three genes (LOC107815210, LOC107790046, and LOC107829090) encoding DMR6-like proteins that catalyze SA hydroxylation and acting as suppressors of SA-mediated phytoimmunity [39] were upregulated. The NPR1 and NPR3 genes, which encode the main component of SA signaling and the SA receptor, respectively, were non-DEGs. The canonical markers of SA pathways—two genes encoding acidic PR-1 proteins with an SA-responsive cis-element (AS-1) in their promoter regions [40]—were not induced and one of them (LOC107807833) was even downregulated. Thus, SA-mediated responses are not induced or even repressed during *Pba*-caused infection.

DEGs for 9-cis-epoxycarotenoid dioxygenases (NCEDs) involved in the initial step of *abscisic acid* (ABA) biosynthesis were slightly downregulated (LOC107827644, LOC107799511, LOC107823578, LOC107791323, and LOC107767418), while both up- and downregulated genes were found among DEGs encoding the ABA 8-hydroxylase-like protein, the key enzyme for ABA catabolism. Interestingly, eight genes for ABA receptors PYL2 (LOC107760113), PYL4 (LOC107776401, LOC107816583, LOC107794010, LOC107822698, LOC107788622, and LOC107778387), and PYL8 (LOC107804547) were induced, presumably pointing to the increase in ABA sensitivity. However, the observed changes in the expression of ABA-related genes are not unambiguous enough to determine whether ABA-mediated alterations took place during the infection or not.

The gene expression profile points to the downregulation of hormonal systems mediated by *auxin, cytokinins, gibberellins, and brassinosteroids*. Two auxin-biosynthetic genes encoding amidase 1 (LOC107807935) and indole-3-pyruvate monooxygenase (LOC107791151), as well as many auxin-responsive genes, including Small Auxin Up RNA (SAUR), were downregulated. However, seven DEGs (LOC107764714, LOC107826904, LOC107819938, LOC107824093, LOC107774327, LOC107797063, and LOC107762349) encoding ILR1 auxin–amino acid hydrolases that release an active auxin from its amino acid conjugate [41] were upregulated. Two cytokinin-biosynthetic genes encoding adenylate isopentenyltransferase (ATIPT) were downregulated (LOC107795022 and LOC107814501) as well as the DEGs related to cytokinin signaling. Herewith, DEGs for cytokinin degradation (cytokinin hydroxylase-like encoded by LOC107784803, LOC107763786, and LOC107766411) and inactivation (zeatin O-glucosyltransferase encoded by LOC107769776, LOC107788105, LOC107801620, and LOC107790576) were upregulated. Three genes encoding gibberellin-biosynthetic enzymes (gibberellin 20 oxidases encoded by LOC107803183, LOC107810416, and LOC107816156) were downregulated, while seven DEGs encoding gibberellin-inactivating enzymes (gibberellin 2-beta-dioxygenases encoded by LOC107782042, LOC107780033, LOC107763812, LOC107781790, LOC107792927, LOC107821505, and LOC107794008) were upregulated. DEGs related to brassinosteroid (BR) synthesis encoding delta(24)-sterol reductases (LOC107795958, LOC107785877, and LOC107776792) and delta(7)-sterol-C5(6)-desaturases (LOC107762070 and LOC107763191) were downregulated. In turn, genes for BKI1, which limits the interaction of the BR receptor BRI1 with the co-receptor BAK1 [42], were upregulated (LOC107812778 and LOC107766802). DEGs encoding the components of the downstream BR signaling cascade, i.e., the BR signaling kinases BSK-like (LOC107831422, LOC107780582, and LOC107799113), BZR1-like (LOC107792445), and BIN2-like (LOC107773955 and LOC107801038), were downregulated.

**Figure 3:**
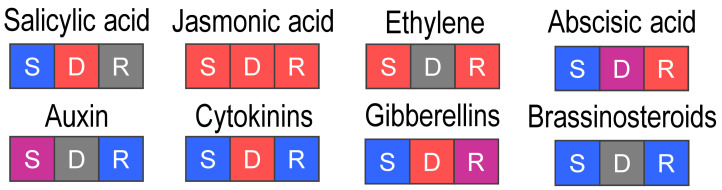
The regulation of *Nicotiana tabacum* differentially expressed genes (DEGs) related to phytohormone metabolism and signaling during *P. atrosepticum*-caused infection. S, genes related to synthesis/activation; D, genes related to degradation/inactivation; R, genes related to receptors/signaling. Red box, most DEGs are upregulated; blue box, most DEGs are downregulated; purple box, both up- and downregulated DEGs are well-represented; grey box, genes are non-differentially expressed during the infection.

Thus, the transcriptome profiling sheds light on the “physiological status” of almost all phytohormonal systems during *Pba*-caused infection (Figure 3).

The only exception is an ABA-mediated one, for which DEGs related to the hormone precursors were repressed, while genes for the hormone receptors were induced. ABA was shown to participate in plant–pathogen interactions, acting mostly as a susceptibility factor but also as a resistance factor [43]. As for SRP-caused pathogenesis, ABA is most likely to serve as a susceptibility factor according to previous studies [44,45].

Growth-related hormonal systems (mediated by auxin, cytokinins, and gibberellins) as well as a BR-mediated one were evidently repressed during *Pba*-caused infection, which is consistent with systemic downregulation of growth processes in the diseased plant. The SA-mediated hormonal system was clearly not induced or even repressed during the disease’s development. SA was definitely shown to promote resistance to SRP when exogenously applied [46,47,48]. Thus, it is evident that the disease would not progress if the SA-mediated hormonal system is induced; therefore, it is “kept in an off state” during soft rot development.

The downregulation of the SA-mediated hormonal system may be determined (at least partially) by the observed induction of JA- and ethylene-mediated ones because of mutual antagonism of SA and JA/ethylene [49,50]. Since JA and ethylene determine the resistance to necrotrophs, these phytohormones are considered to contribute to the resistance to SRP because of the necrotrophic-like mode of action of these pathogens. However, in fact, SRP, like almost all phytopathogenic bacteria, are hemibiotrophs [3], and it is becoming evident that JA does not confer true plant resistance to SRP. A *Pba* mutant deficient in coronafacic acid—a functional analog of JA—is unable to induce plant lipoxygenase gene expression and to cause plant rotting, but retains the ability to colonize plants asymptomatically [35]. Therefore, the systemic induction of the JA-mediated hormonal system revealed by the transcriptome profiling clearly reflects the reaction of induced plant susceptibility that facilitates brute force *Pba* behavior.

The systemic induction of the ethylene-mediated hormonal system observed in our study may be presumed to assist JA-regulated responses since JA and ethylene are known agonists [51]. In addition, some plant cell wall-associated reactions, including those mediated by EXPs and XTHs, are controlled by ethylene [52,53]. In particular, ethylene activates plant cell wall reorganization associated with the tylose formation that is necessary for the manifestation of disease caused by *Xylella fastidiosa* [54]. Therefore, the *Pba*-induced alterations in the expression of plant cell wall-related genes (described above) might be regulated by ethylene.

#### 2.1.3. Primary Metabolism

*Photosynthesis*-related genes were split into three groups according to the patterns of their expression: light-reaction-related and chlorophyll-synthesis-related genes were downregulated, dark-reaction-related genes were non-DEGs, and chlorophyll-degradation-related genes were upregulated.

*Aerobic respiration* was evidently increased in the infected plants since genes related to mitochondrial ETC, the TCA cycle, glycolysis, and the pentose–phosphate pathway were upregulated while anaerobic-respiration-related genes (encoding alcohol dehydrogenases and lactate dehydrogenases) were repressed or non-DEGs. The substrate for the increased respiration at reduced photosynthesis was presumably obtained (at least partially) due to starch catabolism: amylase genes were induced, while starch biosynthetic genes were repressed.

Systemic *transport of carbohydrates* (sucrose) in plants is largely driven by the action of invertases. Many invertase genes were upregulated, while genes encoding invertase inhibitors were mostly downregulated. SWEET transporters are also important participants in systemic carbohydrate transport. Unexpectedly, almost all DEGs encoding SWEET transporters (17 out of 19) were downregulated, including those for SWEET11-like (LOC107791830 and LOC107790569) and SWEET12 (LOC107791385 and LOC107830760), which are involved in sucrose efflux for phloem loading [55]. It has been shown that some pathogens exploit hosts’ SWEET transporters to promote nutrient flow towards themselves [55]. Therefore, it may be assumed that during the infection, the expression of SWEET genes is tightly controlled even with the increased intensity of sugar transport due to the enhanced invertase activity and starch catabolism.

**Figure 4:**
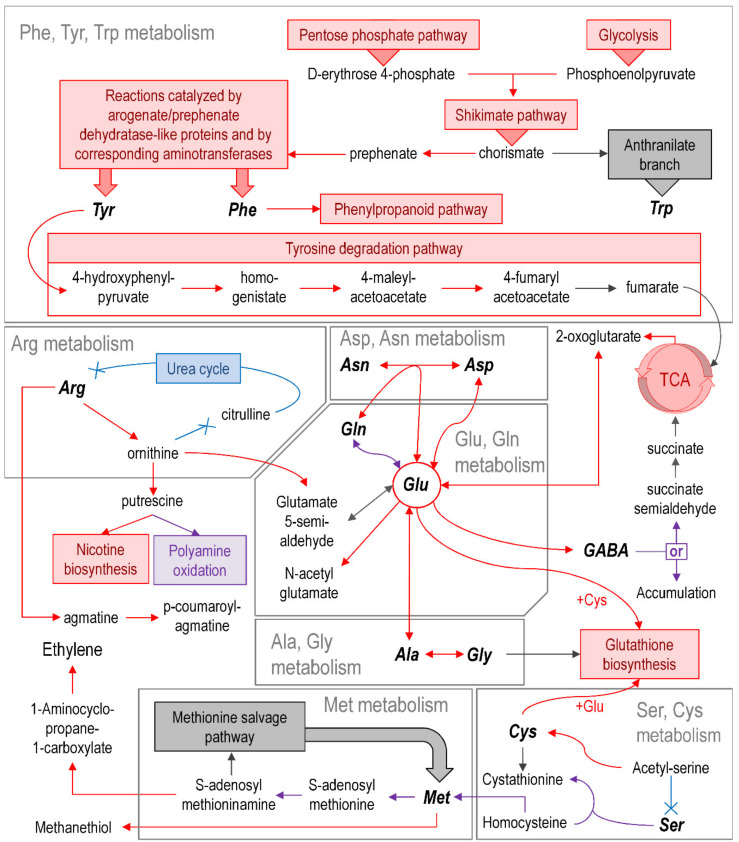
Regulation of genes related to amino acid metabolism in tobacco plants infected by *P. atrosepticum*. Reactions catalyzed by enzymes encoded by mostly upregulated, mostly downregulated, non-DEGs, and both up- and downregulated DEGs are marked in red, blue, gray, and purple, respectively. TCA, tricarboxylic acid cycle. The abbreviated names of amino acids are provided in accordance with the International Union of Pure and Applied Chemistry (IUPAC) recommendations. The scheme was assembled based mostly on Kyoto Encyclopedia of Genes and Genomes (KEGG) reference pathways as well as the SwissProt database and sources of literature cited in the text.

Our RNA-Seq data pointed to a significant reorganization of *amino acid* (AA) metabolism during the infection (Figure 4).

Almost all DEGs related to the synthesis of aromatic AAs were upregulated as well as the DEGs related to the shikimate pathway, which creates a precursor of aromatic AAs (chorismate). Chorismate conversion related to AA metabolism branches into two pathways: the prephenate branch (synthesis of tyrosine and phenylalanine) and the anthranilate branch (synthesis of tryptophan). In our experiments, genes of the prephenate branch were upregulated, while the anthranilate branch genes were non-DEGs. The conversion of Tyr and Phe seemed to be induced since genes related to Tyr conversion to fumarate and genes associated with the synthesis of phenylpropanoids from Phe were upregulated.

An important “hub” of AA metabolism in the infected plants appeared to be glutamate (Glu) since many genes related to its metabolism were upregulated. Upregulated genes for Glu-biosynthetic enzymes included those encoding: (1) glutamate synthases (amination of 2-oxoglutarate) (LOC107767943 and LOC107822887); (2) glutamine-hydrolyzing asparagine synthetases (transamination of glutamine) (LOC107790669, LOC107815637, LOC107794767, LOC107830560, and LOC107770738); (3) alanine aminotransferases ALAAT2-like (transamination of alanine) (LOC107821527 and LOC107800103); and (4) aspartate aminotransferases (transamination of aspartate) (LOC107825707, LOC107800136, and LOC107763688). Genes related to the conversion of Glu to (1) N-acetylglutamate (NAG) (N-acetylglutamate synthase NAGS1) (LOC107769266, LOC107822218, and LOC107804156), (2) gamma-aminobutyric acid (GABA) (glutamate decarboxylase) (LOC107794144 and LOC107819266), and (3) glutathione (glutamate–cysteine ligase GSH1) (LOC107784172) were also upregulated. The latter two (GABA and glutathione) are well-known to participate in the plant stress response. Glutathione is a canonical antioxidant that was shown to be involved in pathogenesis [56]. GABA was also demonstrated to accumulate in plants under a variety of stress conditions and to induce resistance to pathogens [57,58].

Several upregulated DEGs related to arginine (Arg) conversion to ornithine (arginase, LOC107761927, LOC107817319, and LOC107770139) and agmatine (Arg decarboxylase, LOC107759371, LOC107774919, LOC107788017, and LOC107796783) were revealed. Genes associated with ornithine conversion to putrescine, a precursor of nicotine, were upregulated too (LOC107815922 and LOC107801491), which is consistent with the induction of nicotine-related genes (see below). Genes for the agmatine coumaroyltransferase ACT (LOC107761507, LOC107761506, and LOC107794911) that produces p-coumaroylagmatine (CouAgm) were also upregulated. CouAgm was previously shown to be involved in the defense response of *Arabidopsis thaliana* to *Alternaria brassicicola* [59]. In addition, *AtACT* gene expression and CouAgm biosynthesis were reported to be responsive to simultaneous activation of the JA/ET signaling pathways [60], which also took place during the plant–*Pba* interaction (see above).

Around 40 DEGs were related to the metabolism of sulfur-containing amino acids. Genes encoding cysteine synthases (LOC107793495, LOC107788814, and LOC107795843) that catalyze the formation of cysteine (Cys) from acetyl-serine were induced. In turn, it is likely that Cys was spent on glutathione synthesis since genes for cysteine desulfurases were upregulated. Unexpectedly, except for the only homocysteine S-methyltransferase gene (LOC107798163), we have not revealed the upregulation of genes related to methionine (Met) synthesis (via homocysteine or the methionine salvage pathway). Herewith, genes related to several pathways for which Met is a precursor were strongly upregulated. Genes for methionine gamma-lyases (LOC107767855, LOC107818807, LOC107759737, and LOC107799551) that catalyze the formation of methanethiol—a volatile shown to be emitted by *Brassica* spp. in response to herbivores [61]—were upregulated. In addition, genes related to the sequential stages of ethylene biosynthesis from S-adenosylmethioninamine (a product of Met conversion) were strongly upregulated.

**Figure 5:**
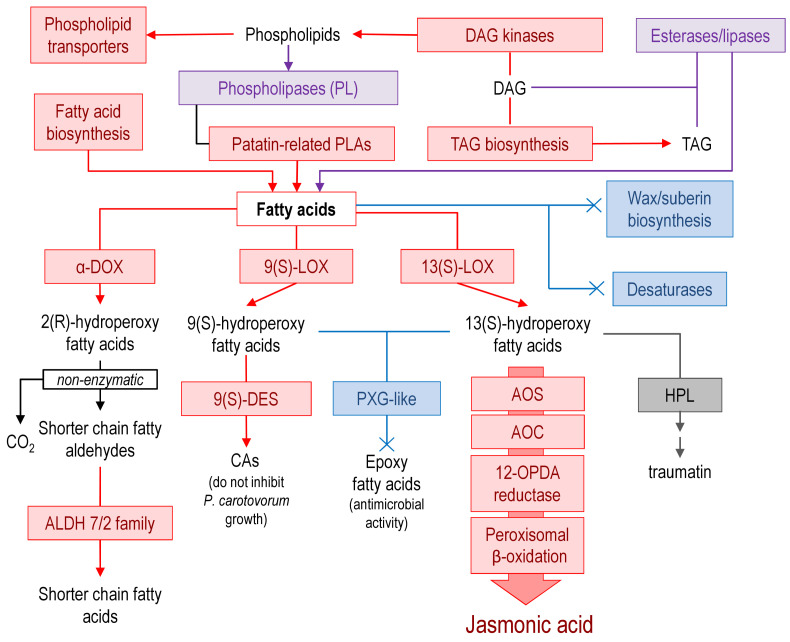
Regulation of genes related to the metabolism of lipids and oxylipins in tobacco plants infected by *P. atrosepticum*. The reactions catalyzed by the enzymes encoded by mostly upregulated, mostly downregulated, non-DEGs, and both up- and downregulated DEGs are marked in red, blue, gray, and purple, respectively. ALDH, aldehyde dehydrogenase; PLA, phospholipase A; α-DOX, α-dioxygenase; CAs, colneleic and colnelenic acids; DAG, diacylglycerol; TAG, triacylglycerol; 12-OPDA, 12-Oxo-phytodienoic acid; PXG, peroxygenase; LOX, lipoxygenase; AOS, allene oxide synthase; AOC, allene oxide cyclase; DES, divinyl ether synthase; HPL, hydroperoxide lyase. The scheme was assembled based mostly on KEGG reference pathways as well as the SwissProt database and sources of literature cited in the text.

The metabolism of *lipids*, in general, was likely to be enhanced during the infection (Figure 5).

Most of the DEGs related to phospholipid and fatty acid synthesis were upregulated except for the plastid monogalactosyldiacylglycerol (LOC107814929, LOC107778411, LOC107796922, and LOC107758934) and digalactosyldiacylglycerol (LOC107801677 and LOC107762360) synthase genes, which were downregulated. Genes encoding fatty acid desaturases were repressed as well as genes associated with wax/suberin biosynthesis. As for the lipid degradation, DEGs encoding phospholipases were equally represented by up- and downregulated genes. However, three of the most upregulated (log_2_FC > 10) phospholipase genes for patatin-related phospholipase A (LOC107759644, LOC107803363, and LOC107771410) were characterized by high expression levels (TPM values) that exceeded by more than 10 times the TPM values for other phospholipase genes, pointing to an increase in lipid degradation. Fatty acids released due to the action of phospholipases may serve as the substrate for lipoxygenases (discussed above in the “Phytohormones” section) and alpha-dioxygenases, genes of which were upregulated during the infection.

#### 2.1.4. Secondary Metabolism

**Figure 6:**
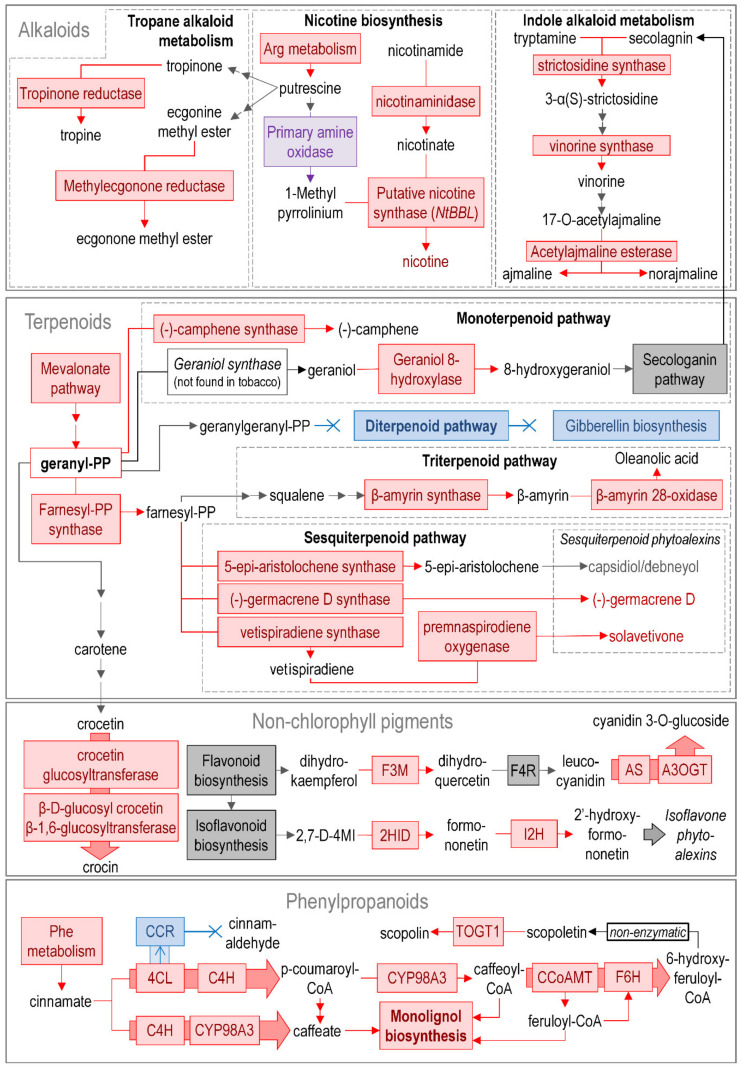
Regulation of genes related to the secondary metabolism in tobacco plants infected by *P. atrosepticum*. The reactions catalyzed by the enzymes encoded by mostly upregulated, mostly downregulated, non-DEGs, and both up- and downregulated DEGs are marked in red, blue, gray, and purple, respectively. F3M, flavonoid 3′-monooxygenase; F4R, flavanone 4-reductase; AS, anthocyanidin synthase; A3OGT, anthocyanidin 3-O-glucosyltransferase; 2,7-D-4MI, 2,7-dihydroxy-4′-methoxyisoflavanone; 2HID, 2-hydroxyisoflavanone dehydratase; I2H, isoflavone 2′-hydroxylase; 4CL, 4-coumarate-CoA ligase; C4H, cinnamate-4-hydroxylase; CCR, cinnamoyl-CoA reductase; CCoAMT, caffeoyl-CoA O-methyltransferase; F6H, feruloyl-CoA 6-hydroxylase; TOGT1, scopoletin glucosyltransferase. The scheme was assembled based mostly on KEGG reference pathways as well as the SwissProt database and sources of literature cited in the text.

Around 350 DEGs were attributed to the “Secondary metabolism” category, which was additionally subdivided into the subcategories “Alkaloids”, “Terpenoids”, “Non-chlorophyll pigments”, and “Phenylpropanoids” (Figure 6).

Some DEGs related to the biosynthesis of different *alkaloids* (of the indole and tropane types) were upregulated during the infection. In addition, strong upregulation was observed for four genes (LOC107771115, LOC107795578, LOC107800217, and LOC107820900) encoding the reticuline oxidase-like protein (syn. NtBBL, berberine bridge enzyme-like protein), which is a putative nicotine synthase [62]. Genes for nicotinamidases (LOC107827940, LOC107820986, LOC107818522, LOC107769430, and LOC107812261) involved in the formation of nicotinate (a nicotine precursor) were also upregulated.

The mevalonate pathway, which yields a diverse range of *terpenoids,* was upregulated in terms of the transcript level. Genes related to a part of the monoterpenoid branch (upstream of the formation of 8-hydroxygeraniol) were upregulated, while genes encoding the enzymes that catalyzed the downstream reactions were mostly non-DEGs. Presumably, this means that 8-hydroxygeraniol plays a significant role in *Pba*-infected plants. In turn, genes encoding the enzymes of diterpenoid biosynthesis were downregulated. This is consistent with the repression of gibberellin-related genes (see above) since the diterpenoid branch yields the precursors of this phytohormone. Genes (LOC107819508, LOC107821306, LOC107773209, LOC107776438, LOC107762781, LOC107762202, LOC107770515, and LOC107760461) related to the synthesis of triterpenoid oleanolic acid (having antimicrobial properties [63]), as well as numerous genes related to the biosynthesis of certain sesquiterpenoids (vetispiradiene, solavetivone, germacrene D, and 5-epi-aristolochene), were upregulated. Germacrene D and solavetivone are phytoalexins [64], while 5-epi-aristolochene is a precursor of the phytoalexins capsidiol and debneyol. Genes related to the final steps of capsidiol and debneyol synthesis were non-DEGs.

The expression of genes related to the synthesis of *non-chlorophyll pigments* was not altered during the infection. Herewith, DEGs related to flavonoid and carotenoid modification were revealed. A strong upregulation was observed for genes (LOC107783555, LOC107761722, LOC107820493. LOC107778878, LOC107790993, LOC107827640, LOC107820494, LOC107763569, LOC107767710, and LOC107780597) encoding biosynthetic enzymes of crocin—a water-soluble apocarotenoid with high antioxidant activity [65]. Genes encoding isoflavone 2’-hydroxylases (yielding different compounds with antioxidant activity [66]) (LOC107782936, LOC107768643, LOC107786246, and LOC107786217) and flavonoid 3’-monooxygenases (LOC107768777, LOC107832265, LOC107827411, LOC107774910, and LOC107783864) were upregulated. The latter enzyme catalyzes the formation of dihydroquercetin—a lipophilic compound with antioxidant properties [67]. Dihydroquercetin can be converted to cyanidin 3-O-glucoside (a water-soluble plant antioxidant also known as chrysanthemin [68]) by the consequent action of flavanone 4-reductase, anthocyanidin synthases (LOC107787195, LOC107795747, LOC107787193, and LOC107808500), and anthocyanidin 3-O-glucosyltransferases (LOC107767027, LOC107811171, LOC107827394, LOC107795162, LOC107798763, and LOC107804884); the listed genes encoding anthocyanidin synthases and anthocyanidin 3-O-glucosyltransferases were upregulated during the infection.

The subcategory “*Phenylpropanoids*” was widely represented by upregulated genes, particularly the branch related to caffeate metabolism. Caffeate and its derivatives are precursors of monolignols and phytoalexin scopoletin. Genes related to the biosynthesis of both monolignols and scopoletin were also induced in our study.

One of the most pronounced levels of induction was found for genes encoding cannabidiolic acid (CBD) synthase-like proteins (LOC107763225, LOC107787464, LOC107829246, and LOC107781064) and the tetrahydrocannabidiolic acid (THC) synthase-like protein (LOC107780227). There is no currently available information about CBD or THC in tobacco plants. Furthermore, despite the fact that cannabinoids have been studied in terms of their pharmacological activity [69,70], the function of these compounds in planta remains uncertain. Our data indicate that cannabinoids are likely to play a significant role in plant-*Pba* interaction.

Thus, our analysis points to the reorganization of the secondary metabolism in *Pba*-infected plants towards the synthesis of the particular compounds with antioxidant (crocin, dihydroquercetin, cyanidin 3-O-glucoside) and phytoalexin (scopoletin, germacrene D, solavetivone, oleanolic acid) properties and the metabolites, whose functions in planta remain to be elucidated (nicotine, 8-hydroxygeraniol, cannabinoids).

#### 2.1.5. Stress-Related Genes

We found 203 DEGs related to *ROS metabolism*. Four of the five DEGs encoding the respiratory burst oxidase homolog protein (RBOH) were upregulated (LOC107785464, LOC107823725, LOC107774415, and LOC107805462). DEGs for other ROS-producing enzymes were represented more or less equally by up- and downregulated genes. According to the transcript profile, the action of RBOH seems to be the major source of ROS during *Pba*-caused infection. This is in accordance with a previous study showing that oxidative burst in *Dickeya*-infected Arabidopsis is mainly generated via the expression of *AtRBOH* independently of the SA-mediated responses [71]. A major portion of the DEGs related to antioxidant systems encodes glutathione metabolic enzymes. Genes for glutathione peroxidases and glutathione reductases were mostly upregulated. In addition, 35 upregulated genes encoding glutathione S-transferases—the enzymes involved in the detoxification of xenobiotics [72]—were revealed. In contrast, genes for ascorbate peroxidases/oxidases were mostly downregulated.

ROS are well-known to mediate *programmed cell death* (PCD), which is represented in plants by autophagy-related PCD, apoptotic-like PCD, and hypersensitive response. *Pba* is known to be unable to induce a hypersensitive response. The other forms of PCD were not analyzed in plants during SRP-caused infection. In our study, 11 DEGs related to apoptotic-like PCD (mainly encoding metacaspases) were almost equally represented by up- and downregulated genes. In turn, seven genes for autophagy-related proteins (ATG) were induced during the infection, and none of the ATG genes were repressed, indicating that autophagy was likely to be involved in plant–*Pba* interactions.

Genes for some *PR proteins* displayed altered expression during the infection. Four genes encoding basic PR-1 proteins (LOC107799202, LOC107768378, LOC107768379, and LOC107798618) were highly upregulated (log_2_FC > 6) and two of them had very high transcript levels (the TPM value was 3081 for LOC107798618 and 4024 for LOC107768378). Genes for the PR-4 chitinase-like protein (LOC107766311) and STH-2-like proteins homologous to PR-10 ribosome-inactivating proteins (RIPs) with RNAse activity (LOC107830186, LOC107823076, LOC107770326, LOC107770507, and LOC107830187) were also characterized by high TPM values (around 1000) and a high degree of upregulation. PR-5 genes were represented by both up- and downregulated ones. Herewith, genes for PR-5 osmotin-like proteins were highly induced and had high expression levels (LOC107814286, LOC107794477, LOC107787819, LOC107794479, LOC107787802, LOC107787812, and LOC107794478), while almost all of the PR-5 thaumatin-encoding genes were repressed.

Several both up- and downregulated DEGs that encode *MLO-like* proteins are known to be susceptibility genes, whose knockout in barley led to resistance to *Blumeria graminis* [73]. Interestingly, among the upregulated MLO genes were those encoding MLO-like protein 6 (LOC107772200, LOC107791015, LOC107764621, and LOC107808566), while genes for MLO-like protein 4 (LOC107815735), MLO-like protein 11 (LOC107822027), and MLO-like protein 13 (LOC107824275 and LOC107792902) were repressed. Changes in the transcriptional profile were also found for 45 genes encoding heat shock proteins (Hsps). Among them, the most represented upregulated genes were those for the chloroplastic chaperone protein dnaJ homologues (e.g., LOC107817371, LOC107794735, LOC107796659, LOC107764029, and LOC107801540) that were previously shown to enhance tomato plant tolerance to *Pseudomonas solanacearum* [74]. In turn, genes encoding Hsps of molecular weight lower than 30 kDa (LOC107786233, LOC107797085, LOC107777821, LOC107810534, LOC107769141, LOC107765678, LOC107790339, LOC107823178, and LOC107821898) were downregulated.

#### 2.1.6. Signaling

We attributed 1324 DEGs encoding various receptors, signaling kinases, G-proteins, Ca^2+^ transporters and sensors, and ubiquitin ligases to the category “signaling”. Many DEGs encode the receptor kinases (RKs) and *receptor-like kinases* (RLKs) recognizing the pathogen-associated molecular pattern (PAMP). DEGs for LysM domain RKs were mostly upregulated, including five genes for CERK1-like (LOC107810925, LOC107782899, LOC107785015, LOC107815265, and LOC107762745) and two genes for LYK4 (LOC107812032 and LOC107788177). CERK1 and LYK4 recognize microbe-derived N-acetylglucosamine-containing ligands, i.e., chitooligosaccharides and peptidoglycan fragments [75]. Forty-eight upregulated DEGs encode G-type lectin RLKs, including those reported to participate in the perception pathogen-derived oligomannosides and high-mannose N-glycans [76]. Herewith, DEGs related to flagellin perception, i.e., genes encoding the FLS2 receptor-like kinase (LOC107814347 and LOC107825117), were downregulated. These results are consistent with our previous data showing that *Pba* genes related to the flagellar apparatus are downregulated during tobacco plant colonization [35]. Therefore, both flagellin synthesis by *Pba* and the sensitivity to flagellin of *Pba*-infected tobacco plants are simultaneously decreased during the infection. As for the perception of the damage-associated molecular pattern (DAMP), namely oligogalacturonides, DEGs encoding wall-associated kinases (WAKs) were mostly upregulated. Most of the DEGs encoding receptor kinases related to growth and development (BAM, CLAVATA, HAIKU, IMK, IRK, TDR, and PXC) were downregulated. The exceptions were the HSL kinases involved in floral organ abscission, for which one-half of the DEGs were upregulated (LOC107766257, LOC107797502, LOC107798498, LOC107762879, LOC107804687, LOC107778576, LOC107777593, LOC107774291, and LOC107765352).

*Pba* is known to be unable to induce effector-triggered immunity (ETI) mediated by plant *R genes*. However, despite the fact that many R genes were downregulated during the infection, we revealed a large group of upregulated ones that encoded late blight resistance R1-like proteins.

Genes encoding the components of the *MAP-kinase* cascade—the crucial “player” in many signal-transmitting events—were also found among the DEGs. However, among the genes for those MAPKs that are known to be involved in plant immunity (MAPK3, MAPK4, and MAPK6), only one (MAPK3), which acts as an activator of the ethylene-responsive transcription factor ERF1, was upregulated (LOC107821857). In addition, two genes for MAPKK9 (LOC107765244 and LOC107819291) acting upstream of MAPK3 were induced. The largest group of DEGs among the upregulated MAPK-cascade-related genes encoded MAPKKK YODA. However, genes for the LRR receptor kinase ERECTA acting upstream of YODA and hypothesized to play a role in plant immunity [77] were not induced during the infection; the same was found for the genes for MAPKK4/5 acting downstream of YODA. Four DEGs encoding MAPKKK NPK1 (LOC107782983, LOC107817248, LOC107828043, and LOC107777428), involved in the regulation of virus-induced ETI [78], were upregulated.

A number of the revealed DEGs encode *Ca^2+^-dependent protein kinases* (CDPKs) that are well-known to participate in the biotic stress response [79]. Most of the CDPK-encoding DEGs were upregulated during *Pba*-caused infection. DEGs for another Ca^2+^ sensor, calmodulins, were almost equally represented by up- and downregulated genes, pointing to alterations in the calmodulin repertoire during the infection. DEGs related to Ca^2+^ influx were mostly upregulated. DEGs encoding glutamate-gated non-selective cation channels (glutamate receptors, GRLs) presumed to act as Ca^2+^ transporters from apoplast [80] were mostly upregulated (LOC107797329, LOC107790599, LOC107796560, LOC107807265, LOC107812552, LOC107800505, LOC107759566, LOC107759340, and LOC107800506). In addition, DEGs for the tonoplastic cation/Ca^2+^ exchanger CCX1 (LOC107823078 and LOC107764581) and DEGs encoding LETM1-like H^+^/Ca^2+^ exchangers (LOC107823016 and LOC107775562) that provide Ca^2+^ efflux from vacuoles and mitochondria, respectively, to the cytoplasm [81,82] were upregulated. In turn, DEGs that encode Ca^2+^ transporters providing Ca^2+^ flow from the cytoplasm to mitochondria or the endoplasmic reticulum were downregulated (LOC107801276, LOC107759900, LOC107800236, LOC107803970, LOC107800917, LOC107781311, and LOC107781432).

Many DEGs are related to *ubiquitination,* which is referred to as a “signaling hub” because of its crucial role in many signaling events [83]. Most of these DEGs (both up- and downregulated) encode E3 ubiquitin ligases that determine the substrate specificity of ubiquitination. Several groups of DEGs for E3 ligases had especially pronounced upregulation: PUB21-like, PUB23-like, PUB24-like, ATL31-like, and RMA1-like, some of which were shown to play a role in plant immunity [84,85,86]. All DEGs for polyubiquitin were also upregulated. DEGs for E2 ubiquitin-conjugating enzymes were almost equally represented by up- and downregulated genes and no DEGs for E1 ubiquitin-activating enzymes were revealed.

**Figure 7:**
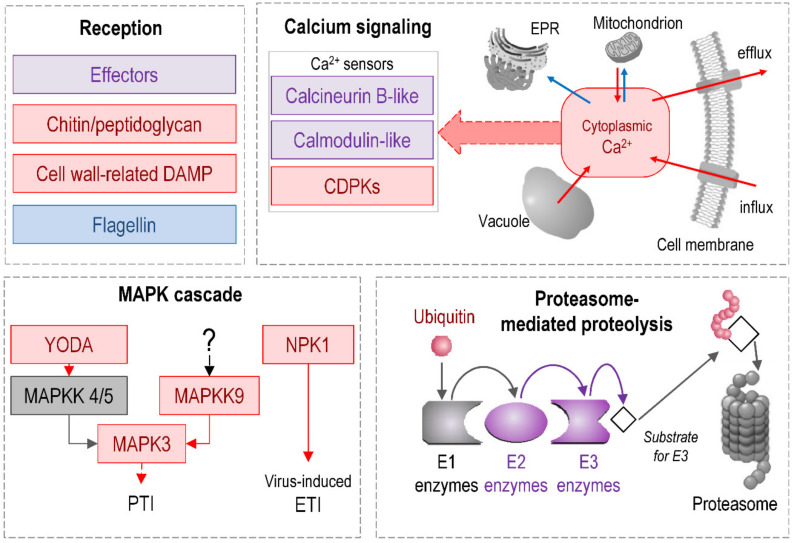
Regulation of genes related to signal perception and transmission in tobacco plants infected by *P. atrosepticum*. Signaling-related proteins encoded by mostly upregulated, mostly downregulated, non-DEGs, and both up- and downregulated DEGs are marked in red, blue, gray, and purple, respectively. The scheme was assembled based mostly on KEGG reference pathways as well as the SwissProt database and sources of literature cited in the text.

Thus, the transcription profile points to significant changes in signaling events during *Pba*-caused infection (Figure 7).

The sensitivity of the infected plants to various PAMPs and DAMPs (except for flagellin) was likely increased due to the upregulation of genes of corresponding receptors. Herewith, R genes are not induced except for the only group encoding late blight resistance R1-like proteins. As for signal transduction machinery, ubiquitination and Ca^2+^-related processes are presumably activated. In turn, we did not find evidence for the systemic induction of genes of well-known signal transmitters—MAPKs—known as “canonical” for biotic stress. This presumably means that the progression of *Pba*-caused disease is coupled with the repression of some aspects of signal transduction events.

### 2.2. Transcription Factors

The transcriptome analysis pointed to a significant change in the transcription factor (TF) profile during the infection: 676 TF-encoding genes were DEGs. DEGs encoding ERF- and WRKY-family TFs were mostly upregulated, while DEGs for bHLH-, DOF-, and GATA-family TFs were mostly downregulated (for details see Supplementary Table S2).

Some TFs, the so-called “master switchers”, are known to control the manifestation of phenotypes determined by a large number of gene products (e.g., plant cell wall structure and properties) [87,88,89]. Such TFs are promising targets for engineering various plant hallmarks. The identification of major disease-related TFs is necessary to decipher the mechanisms of regulation of pathogen-induced plant responses. To identify the TFs potentially involved in *Pba*-induced plant responses based on the RNA-Seq data, we differentially analyzed the expression patterns of genes comprising the predicted regulons of different TFs using the original pipeline. We determined whether a gene belongs to a particular regulon by the presence of the cis-regulatory element (CRE) corresponding to the particular TF in the promoter region (1000 bp upstream of the transcription start site) of a gene. Then, we determined whether the pools of expressed genes regulated by the particular TFs were significantly enriched by DEGs. We used custom scripts (1) to identify CREs and corresponding TFs; (2) to search for the correlation between the presence of the particular CRE and the expression pattern; (3) to visualize the results. The scripts, which are available at https://github.com/IvanTsers/Promoter-analysis, can be easily adapted for use in related studies.

In total, 2,273,233 sites for 1340 annotated variants of CRE motifs (related to 999 transcription factors) were revealed within the 1000-bp-length promoter regions of tobacco genes. Among them, 405,061 sites for 1287 annotated variants of CRE motifs (related to 992 transcription factors) were located in the promoters of DEGs. A statistical test showed that pools of expressed genes regulated by seven TFs of the WRKY (WRKY6, 42, 45, 51, and 57) and TCP (TCP3 and 15) families were enriched by DEGs (Table 1).

Genes encoding some of these TFs, namely those of the WRKY family (WRKY6, 42, 45, and 51), were upregulated. Two DEGs for TCP15 were downregulated, and no expressed genes for TCP3 were revealed. Thus, WRKY6, 42, 45, and 51 are the top candidates for master switchers involved in the regulation of *Pba*-induced plant responses; genes encoding these TFs were upregulated, and pools of their target genes were enriched by DEGs. Herewith, TCP3 and TCP15 are also likely to play important roles in *Pba*-caused pathogenesis; although TCP3- and TCP15-encoding genes were not induced, the regulons of these TFs were enriched with DEGs. Therefore, it is possible that we observed only the consequences of TCP3 and TCP15 gene induction that might have taken place at earlier infection stages than the analyzed one.

**Figure 8:**
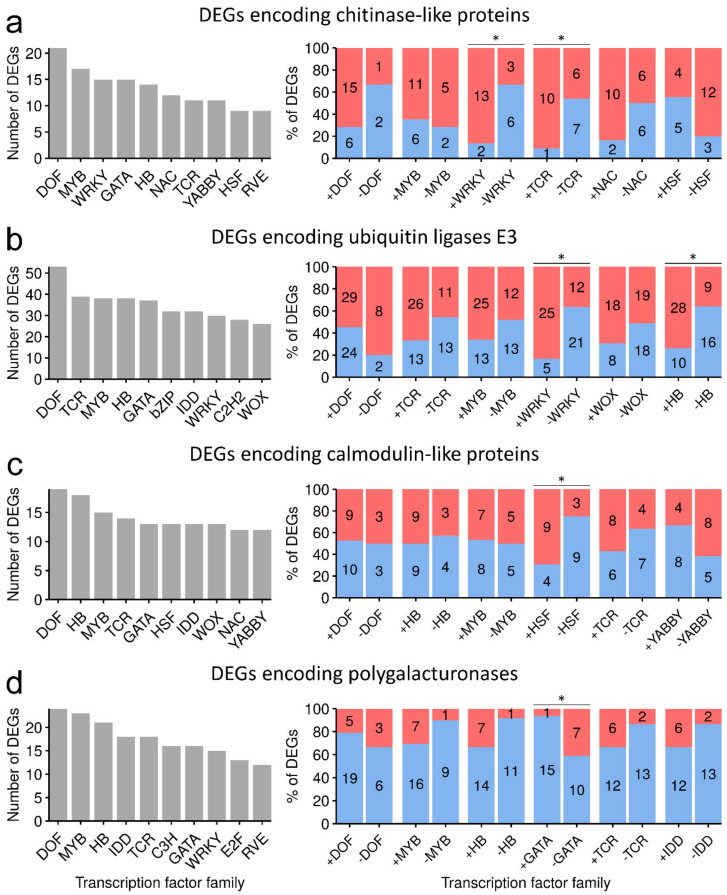
The effect of cis-regulatory elements (CREs) related to different transcription factors in the promoter regions of genes encoding chitinase-like proteins (**a**), ubiquitin E3 ligases (**b**), calmodulin-like proteins (**c**), and polygalacturonases (**d**) on their expression pattern. Left-hand side panels: the total number of DEGs with CREs attributed to the TF families denoted on the X-axis (the top 10 TF families are presented). Right-hand side diagrams: the proportions of up- (red) and down- (blue) regulated DEGs (%) depending on the presence (+TF) or absence (-TF) of CREs for a certain TF family. The numbers in the columns show the absolute number of DEGs in each category. Data for six groups of TFs are presented: the first three pairs of columns are for the top three TF families from the left bar chart; and the next three pairs of columns are for those TF families for which the differences in the ratios of up/downregulated target DEGs and the ratios of up/downregulated non-target DEGs are the highest. Asterisks (*) show the significance of the difference (*p* < 0.05) in the ratios of up/downregulated genes between the groups of DEGs with and without CREs for TFs of a certain family. The significance was determined using Fisher’s exact test.

A distinctive feature of a plant genome is the presence of especially large multigene families that include genes encoding cognate proteins. Herewith, genes of the same multigene family can be equally represented by the induced and repressed ones, and the reasons for and essence of such gene regulation are often hard to interpret. We hypothesized that a split in the expression pattern of genes belonging to the same multigene family into induction and repression might have taken place due to their regulation by different TFs because of the presence or absence of certain CREs in the promoters of these DEGs. To check this hypothesis, we analyzed if the expression pattern of the induced and repressed genes encoding cognate proteins depends on whether a gene belongs to the predicted regulon of a certain group of TFs or not. This analysis was performed by the example of groups of genes encoding chitinase-like proteins, polygalacturonases, calmodulin-like proteins, and ubiquitin E3 ligases because large portions of the genes encoding these proteins were DEGs that were well-represented by both up- and downregulated ones. For a given gene group, the percentage ratio of up/downregulated genes was calculated differentially for those genes that belong to the predicted regulon of a certain TF family (i.e., have corresponding CREs) and those genes that are not regulated by these TFs (i.e., do not have corresponding CREs) (Figure 8). Then, we assessed if the ratios of up/downregulated genes are significantly different between the groups of genes with and without CREs for TFs of a certain family.

The ratios of up/downregulated chitinase-encoding DEGs were significantly different in the groups of the DEGs with and without CREs for WRKY- and TCR-family TFs (Figure 8a); significantly more chitinase-encoding DEGs having these CREs were upregulated than those that did not have them. This means that those chitinase-encoding genes that are regulated by WRKY- and/or TCR-family TFs are preferentially induced during *Pba*-caused infection. The presence of WRKY-related CREs in the promoters of ubiquitin E3 ligase-encoding genes also seemed to contribute to their upregulation; the same can be said for the presence of CREs for Homeobox-leucine zipper protein (HB) TFs (Figure 8b). These data are in agreement with our results showing that WRKY-family TFs are top candidate master switchers during *Pba*-caused infection (Table 1). In addition to WRKY-family TFs, TCR- and HB-family TFs contributed to the determination of the spectrum of upregulated genes encoding chitinases and ubiquitin E3 ligases, respectively. For calmodulin-encoding genes, those that have CREs for heat shock factor (HSF) TFs were preferentially upregulated compared with those that did not have them (Figure 8c). As for polygalacturonase-encoding genes, we did not find the TFs that contributed to their upregulation, but, herewith, we revealed that the presence of CREs for GATA-family TFs was a probable cause of downregulation of polygalacturonase genes (Figure 8d).

Thus, our results reveal candidate master switchers (WRKY6, 42, 45, 51, and 57 and TCP3 and 15) that presumably play prominent roles in the regulation of *Pba*-induced plant responses. The pools of target genes of these TFs were demonstrated to be enriched with DEGs. We have also shown that one of the reasons for a split of the expression pattern of DEGs encoding cognate proteins into induction and repression might be the presence or absence of certain CREs recognized by different TFs in the promoters of these DEGs. Based on RNA-Seq data, WRKY-, TCR-, HB-, and HSF-family TFs were predicted to specifically determine the spectrum of up- or downregulated DEGs belonging to the particular multigene family.

## 3. Materials and Methods

### 3.1. Bacteria and Plant Growth Conditions, Plant Inoculation, and Sample Collection

A strain of *Pectobacterium atrosepticum* SCRI1043 (*Pba*) (ATCC accession number BAA-672), kindly provided by Dr. Yevgeny A. Nikolaichik (Belarus State University, Minsk, Belarus), was grown overnight in Luria–Bertani (LB) broth on a rotary shaker (180 rpm) at 28 °C. *Nicotiana tabacum* cv. Petit Havana SR1 plants were grown axenically in test tubes placed in a growth chamber with a 16-h light/8-h dark cycle photoperiod. Seeds were surface-sterilized using diluted bleach (0.8% of active chlorine) and 1% sodium dodecyl sulfate for 30 min, washed seven times with sterile distilled water, then transferred to Murashige and Skoog medium (MS) in Petri dishes. Ten-day-old seedlings were transferred to individual flasks containing MS.

Six to seven weeks after planting, tobacco plants were infected with *Pba*. For plant inoculation, bacteria were grown until the early stationary phase (~2 × 10^9^ colony-forming units, CFU mL^−1^), then washed with sterile 10 mM MgSO_4_ and resuspended in the same solution up to a density of ~2 × 10^7^ CFU mL^−1^. Sterile 10 mM MgSO_4_ or bacterial suspensions containing ~2 × 10^5^ cells were placed as 10 μL drops into the bosoms of the leaves in the middle part of the stems using sterile pipette tips and slight scratches were made simultaneously. Two days after plant inoculation, when disease symptoms were visible, stem sections in the asymptomatic stem zone (1 cm below the symptomatic, macerated area formed at the point of inoculation) were taken from the infected plants for RNA extraction; the corresponding stem sections were taken from non-infected plants for control.

### 3.2. RNA Extraction and cDNA Library Preparation

Plant material was ground in liquid nitrogen in mortars. The obtained powder was resuspended in 1 mL of ExtractRNA Reagent (Evrogen, Moscow, Russia) and the subsequent procedures were performed according to the manufacturer’s instructions. Residual DNA was eliminated using a DNA-free kit (Life Technologies, Carlsbad, USA). RNA quantity was analyzed using a Qubit fluorimeter (Life Technologies, Carlsbad, USA).

For RNA-Seq, total RNA (3 μg) was processed using a Ribo-Zero rRNA Removal Kit (Plant) (Illumina, San Diego, USA) and then a ScriptSeq™ Complete Kit (Bacteria)—Low Input (Illumina, San Diego, USA), which included a bacterial rRNA depletion step, according to the manufacturer’s instructions. The quality and quantity of the cDNA libraries during processing before sequencing were monitored using an Agilent 2100 Bioanalyzer (Agilent, Santa Clara, USA) and a CFX96 Touch Real-Time PCR Detection System (Bio-Rad, Hercules, USA). Libraries were sequenced in two biological replicates. Sequencing was conducted on an Illumina HiSeq 2500 platform (Illumina, San Diego, USA) at the Joint KFU–Riken Laboratory, Kazan Federal University (Kazan, Russia).

### 3.3. Identification and Classification of DEGs

Raw read datasets can be accessed from the NCBI’s BioProject under the accession number PRJNA636273. The quality of the obtained reads was assessed using the FastQC tool (http://www.bioinformatics.babraham.ac.uk/projects/fastqc/). Bad-quality bases of reads (*q*-score < 30) and short ( < 40 bp) reads were removed from the libraries. All reads were trimmed to a maximum length of 58 bp to remove the 3′-end sequencing artifacts. Read filtering and trimming were carried out using Trimmomatic [90] with a custom parameter set (SE CROP:58 MINLEN:40 AVGQUAL:30). rRNA-corresponding reads were filtered out using the SortMeRNA tool and standard reference rRNA databases [91]. To determine whether the RNA-seq data were self-consistent in biological replicates within the control and experimental sample groups, the hierarchical clustering of Euclidean distances between the replicates in terms of TPM values was performed using the hclust function in R.

The EvidentialGene package (https://sourceforge.net/projects/evidentialgene/) was used with default parameters to reduce the redundancy of tobacco CDS (*N. tabacum* reference assembly, NCBI Assembly accession GCF_000715135.1) in order to create the optimal reference for performing the read pseudo-alignment. Pseudo-alignments of filtered reads and transcript quantification were carried out using kallisto [92] with default parameters. The edgeR package [93] was used to reveal differentially expressed genes (DEGs). Genes that had CPM ≥ 1 for all replicates within at least one of the experimental conditions (infected or control plants) were considered to be expressed genes in our study. Genes with |log_2_FC| > 1 and FDR < 0.05 were considered to be DEGs.

To carry out detailed gene sorting for interpretation of RNA-Seq data in terms of general physiology, the following steps were made. NCBI RefSeq annotations of tobacco genes were fused to our RNA-Seq data. Genes annotated in the *N. tabacum* reference assembly as genes encoding the unknown proteins were additionally annotated based on their similarity to *Arabidopsis thaliana* proteins using BLAST+ (with a 1 e^-7^ e-value threshold cut-off), and TAIR10 annotations were fused to our RNA-Seq data. Then, the automatic classification of DEGs into functional categories was performed using KEGG database, Mercator [94], and MapMan [95] software, and the CAZy (http://www.cazy.org/) database. The merged classification based on the above-mentioned databases was created, manually checked, edited, and enriched by the “missed” genes based on the information from the SwissProt database and numerous sources of literature. Gene categories were manually subdivided into different subcategories (e.g., the “Cell wall” category was divided into “Cellulose synthesis”, “Cross-linking glycan (CLG) synthesis”, “CLG degradation”, “Polygalacturonan (PG) synthesis”, “PG degradation”, “Rhamnogalacturonan (RG) synthesis”, “RG-degradation”, “Callose synthesis”, “Callose degradation”, “Lignin synthesis”, and “Cell wall proteins”). The subcategories were additionally split into lower ranks that include genes for the particular enzymes/proteins (e.g., the “CLG degradation” subcategory was divided into “Arabinofuranosidases”, “Endoglucanases”, “Glucosidases”, “Glucosidases”, “Xyloglucan endotransglycosylases/hydrolases”, and “Xylosidases/xylanases”). The original DEG classification is presented in supplementary Table S2.

The schematic representation of regulated pathways or functional gene groups (Figure 3, Figure 4, Figure 5, Figure 6 and Figure 7) was made using standard Microsoft office software. Pathways or functional gene groups were assembled based mostly on KEGG reference pathways as well as the SwissProt database and sources of literature cited in the text. The type of regulation was assigned to the pathways or gene groups based on the corresponding gene expression pattern (log_2_FC and TPM values) and the role of corresponding gene products in a given pathway (e.g., if gene products catalyzed the synthesis of distant or close precursors of the target metabolites, if gene products were positive or negative regulators, etc.). Red, blue, gray, and purple colors indicate that the pathway or functional gene group (according to the gene expression pattern) was upregulated, downregulated, not regulated, or characterized by undetermined regulation (represented by the products of both up- and downregulated genes rather equally). The schemes summarize the information discussed in the text.

### 3.4. Prediction of the Transcription Factors Involved in Regulation of Pba-Induced Plant Responses

The following steps were made to reveal the potential master regulator TFs of *Pba*-induced plant responses and TFs that might determine the expression pattern (up- or downregulation) of genes belonging to the same multigene family, within which both up- and downregulated genes were well-represented. To search for cis-regulatory elements (CREs) within promoter regions of tobacco genes, sequences located up to 1000 bp upstream of the transcription start sites were extracted using an open-source Bash shell script (https://github.com/RimGubaev/extract_promoters). Position weight matrices (PWMs) for the CRE search were obtained from the PlantPAN 3.0 database [96]. Scanning for CREs was performed using the Motif Alignment and Search Tool (MAST) [97]. Since MAST does not support a single file with thousands of PWMs as input, we split a large file with PWMs into 13 files, each with 50 PWMs, that were used as the input into MAST. Thirteen MAST output XML files were converted into a single flat file table using the R package’s XML parser [98]. This table contained gene IDs with fused information about CREs (PWM IDs in the PlantPAN3.0 database, names of transcription factors related to CREs, and names of the respective TF families).

The expressed genes were split into different pools (predicted TF regulons) based on the presence of a particular CRE in the promoter region. Then, we calculated the numbers of genes for each predicted regulon and tested if any regulons were enriched by DEGs. The significance of DEG enrichment was estimated using the hypergeometric test, and *p*-values were adjusted with the Benjamini and Hochberg procedure (FDR). The predicted regulons for which the test yielded an FDR < 0.05 were considered to be significantly enriched with DEGs, and TFs corresponding to DEG-enriched regulons were considered to be the potential master regulators.

To check if the expression pattern (up- or downregulation) of genes within a given multigene family (encoding cognate proteins, e.g., chitinase-like proteins, polygalacturonases, ubiquitin E3 ligases, or calmodulin-like proteins) depends on whether a gene is regulated by TFs of a particular family, the following steps were made. CREs related to TFs of a particular family were grouped. DEGs of a particular multigene family were split into two groups based on the presence (+TF) or absence (-TF) of CREs related to TFs of a particular family in the promoters. If multiple CREs related to a particular TF family were present in the promoter of a given gene, only one of the CREs was considered. The ratios of up- and downregulated DEGs in the groups “+TF” and “-TF” were counted. The significance of differences in the ratios of up- and downregulated DEGs in the “+TF” and “-TF” groups was determined by Fisher’s exact test (*p* < 0.05). If the difference was significant, we considered the corresponding TF family to have contributed to the determination of the spectrum of up- or downregulated genes within a multigene family.

### 3.5. Verification of RNA-Seq by qRT-PCR

One microgram of DNAse-treated RNA was used for cDNA synthesis using RevertAid reverse transcriptase (Thermo Fisher Scientific, Waltham, USA) according to the manufacturer’s instructions. Two microliters of 5-fold-diluted cDNA were used for qPCR. qPCR was performed using the EVA-Green-containing master mix (Syntol, Moscow, Russia) according to the manufacturer’s instructions. Primers for target and reference genes (Supplementary Table S2) were designed using Vector-NTI Version 9 software (Thermo Fisher Scientific, Waltham, USA) and synthesized by Evrogen company (Moscow, Russia). Genes encoding elongation factor 1-alpha and ATP-synthase subunit beta, the transcript level of which was confirmed by geNorm software (http://genorm.cmgg.be/) to be stable under the experimental conditions (data not shown), were used for normalization of the target gene expression. Relative expression levels were determined as the ratios between the quantities of cDNA corresponding to the target genes and values of the normalization factor, which was calculated for each sample using geNorm software based on transcript levels of reference genes. The presented data were obtained by the analysis of at least four biological replicates.

### 3.6. Lipoxygenase and Divinyl Ether Synthase Activity Assay

Lipoxygenase and divinyl ether synthase activities were determined by monitoring the increase of the signals at 234 nm (lipoxygenase activity) and 267 nm (divinyl ether synthase activity) using a РВ2201В spectrophotometer (SOLAR, Minsk, Belarus) with linoleic acid as a substrate [99,100]. Plant material was ground in liquid nitrogen in mortars and then homogenized in 3 volumes (w/v) of 0.05 M Na phosphate buffer (pH 7.5). The obtained homogenates were centrifuged (18,000 g, 4 °C, 15 min), and the activity was determined in the supernatants (5 µL per assay). The analyses were performed in 0.6 mL of 0.05 M Na phosphate buffer (pH 7.5) at 25 °C. The initial linear regions of the kinetic curves were used to calculate the rates. The activities were measured in five biological replicates and expressed as U/min/g of fresh weight of plant material.

## 4. Conclusions

In our study, we applied original detailed gene sorting and custom pipelines in order to draw a picture of the physiological events in *Pba*-infected tobacco plants based on RNA-Seq data. Our results strengthen and deepen previously proposed hypotheses about SRP-caused pathogenesis as well as reveal novel aspects of and point to novel molecular “players” in plant–*Pba* interactions. We describe the defense- and susceptibility-related “sides” of plant cell wall modification in terms of gene expression and predict the particular enzymes/proteins that fulfill the reaction of rhamnogalacturonan (RG) release into the vessel lumen, where RG serves as a matrix for bacterial emboli assemblage. Upregulation of genes encoding XTHs, EXPs, polygalacturonases, RG lyases, and beta-galactosidases points to the mechanism of this reaction: XTHs and EXPs provide plant cell wall loosening, while polygalacturonases, RG lyases, and beta-galactosidases split pectic polymers to enable the release of RG fragments from the plant cell wall into the vessel lumen.

Activation of lipid-metabolism-associated genes was demonstrated along with differential regulation of oxylipin-biosynthetic branches; genes of jasmonate- and divinyl-ether-related branches yielding the compounds that are non-toxic for *Pba* are upregulated, while those of the epoxyalcohol-related branch producing potentially toxic metabolites and the traumatin-related branch are not or even repressed. The upregulation of genes related to oxylipin-biosynthetic branches was shown to be associated with the increase in lipoxygenase and divinyl ether synthase activities. These results show that induction of the lipoxygenase pathway (including the jasmonate-related branch) is a hallmark of *Pba*-caused disease. This is supported by the fact that in *Pba*-infected potato plants, jasmonate-related genes are also upregulated. Ethylene (a jasmonate agonist) is also likely to play an important role in *Pba*-caused soft rot development; the expression of ethylene-related genes was induced in both tobacco and potato plants after infection with *Pba*.

The substrate for the explicitly increased aerobic respiration at repressed photosynthesis during the infection is suggested to be provided due to the enhanced transport of assimilates conferred by the revealed upregulation of invertase genes and starch-degradation-related genes. Glutamate is proposed to be an important “hub” of amino acid metabolism that together with arginine and aromatic amino acids (tyrosine and phenylalanine) produces a number of metabolites with antioxidant, phytoalexin, and other defense properties. Secondary metabolism was shown at the gene expression level to be reorganized towards the enhanced synthesis of the compounds with antioxidant (crocin, dihydroquercetin, cyanidin 3-O-glucoside) and phytoalexin (scopoletin, germacrene D, solavetivone, oleanolic acid) properties and metabolites whose functions in planta remain to be elucidated (nicotine, 8-hydroxygeraniol, cannabinoids). RBOH and glutathione were proposed to be the major participants in redox metabolism in *Pba*-infected plants and the induction of autophagy-related genes was revealed during the infection. A plausible increase in sensitivity to PAMPs/DAMPs (except for flagellin sensitivity) of *Pba*-infected plants and the activation of Ca^2+^- and ubiquitination-related signalization were both inferred from the transcriptome profile.

The TFs WRKY6, 42, 45, 51, and 57 and TCP3 and 15 were revealed to be candidate master regulators of *Pba*-induced plant responses since their predicted regulons were shown to be enriched by DEGs. In addition, the regulation type (induction or repression) of a DEG belonging to a particular multigene family well-represented by both up- and downregulated genes was determined (in some cases) by whether a gene belongs to the regulons of TFs of a particular family or not. To the best of our knowledge, multiple screenings of gene expression patterns within regulons have not been carried out by the example of transcriptome-wide studies of plant infections prior to our investigation. As for the infection-unrelated studies, the correlation between the presence of a CRE and the expression pattern was tested only for the particular TFs and/or particular target genes [101,102,103], but not for the whole spectrum of genes and annotated CREs as performed in our study.

## Figures and Tables

**Table 1:** Transcription factors for which the pools of target genes were enriched with genes expressed differentially in non-infected and *P. atrosepticum*-infected tobacco plants. FDR values were obtained using Fisher’s exact test with BH adjustment. The “expression pattern” column shows log_2_FC values for the expression of genes encoding the TFs. Non-EXP stands for non-expressed gene; non-DEG stands for non-differentially expressed gene.

Transcription Factor	Amount of Predicted Target Genes					FDR	GeneID (RefSeq)	Expression Pattern, log_2_FC
	Up Regulated DEGs	Down Regulated DEGs	Total DEGs	Total Non-DEGs				
TCP3	62	101	163		281	0.0405	LOC107768765	nonEXP
TCP15	93	110	203		361	0.0405	LOC107795877	−4.424
							LOC107800433	−2.127
WRKY6	362	281	643		1335	0.0405	LOC107807966	5.332
							LOC107782559	5.427
							LOC107824974	2.742
WRKY42	408	323	731		1505	0.0289	LOC107770788	1.352
							LOC107824355	2.441
WRKY45	463	370	833		1727	0.0289	LOC107763775	5.993
							LOC107769441	7.22
WRKY51	78	51	129		208	0.0405	LOC107787246	2.64
WRKY57	42	31	73		103	0.0405	LOC107815490	nonDEG
							LOC107788682	nonDEG

## Supplementary Materials

The following are available online at https://www.mdpi.com/2223-7747/9/9/1176/s1: Figure S1: The analysis of self-consistency of expression data of biological replicates within the control (non-infected tobacco plants) and experimental (*P. atrosepticum*-infected tobacco plant) groups, Table S1: The details of RNA-Seq read processing and mapping, Table S2: The original classification of DEGs and the sequences of the primers used for DEG verification (a multi-sheet Excel workbook), Supplementary file 1: lipoxygenase-pathway-related and ethylene-regulated gene expression in *Pectobacterium-atrosepticum*-infected potato plants.
