# Supplementary material for: Plant Soft Rot Development and Regulation from the Viewpoint of Transcriptomic Profiling
Source: Plants (Basel). 2020 Sep 10;9(9):1176. doi: 10.3390/plants9091176 (PMC7570247; doi:10.3390/plants9091176)
Supplement: Supplementary file 1 [file plants-09-01176-s001.zip › Figure S1.doc]

**Figure S1.** The analysis of self-consistency of expression data of biological replicates within control (non-infected tobacco plants) and experimental (P. atrosepticum-infected tobacco plant) groups. The dendrogram was calculated using the hierarchical clustering of Euclidean distances between the samples. The distances were calculated using the TPM values.
