# Supplementary material for: Plant Soft Rot Development and Regulation from the Viewpoint of Transcriptomic Profiling
Source: Plants (Basel). 2020 Sep 10;9(9):1176. doi: 10.3390/plants9091176 (PMC7570247; doi:10.3390/plants9091176)
Supplement: Supplementary file 1 [file plants-09-01176-s001.zip › Supplementary file 1.doc]

Supplementary file 1. Lipoxygenase pathway-related and ethylene-regulated gene expression in *Pectobacterium atrosepticum*-infected potato plants.

Virus-free potato (*Solanum tuberosum* cv. Condor) plants were propagated under sterile conditions. Apical parts (2 cm length) of sterile plants were rooted aseptically in vermiculite. Then, the plants were grown in 300 ml plastic pots on commercial soil (Terra Vita, Russia) with a 16-h light/8-h dark cycle photoperiod at 20 °C. Four weeks after planting into the soil, plants were infected by *Pectobacterium atrosepticum* SCRI1043 (*Pba*). Stem sections from *Pba*-infected and mock-treated plants were sampled for RNA extraction two days post inoculation. RNA extraction, cDNA synthesis, and qPCR were carried out as it was described in the “Materials and methods” section.

| **Gene symbol** | **Gene RefSeq ID (*S. tuberosum*)** | **Gene RefSeq ID (*N. tabacum*)** |
| --- | --- | --- |
| JAZ | LOC102580049 | LOC107815661 |
| AOS | LOC102577479 | LOC107767065 |
| LOX | LOC102577615 | LOC107830099 |
| ERF1-like | LOC102587576 | LOC107805280 |
| ChiA-like | LOC102589117 | LOC107759005 |
| Win2-like | LOC102581743 | LOC107794263 |

**Figure S2.** The expression level (Log_2_FC) of jasmonic acid-related (JAZ, AOS, LOX) and ethylene-induced (ERF1-like, ChiA-like, Win2-like) genes in *Pba*-infected potato (white columns) and in *Pba*-infected tobacco (black columns) plants compared to non-infected plants. The assayed genes are homologs in tobacco and potato plants. The expression of potato genes was measured using qPCR. The expression of tobacco genes was measured using RNA-Seq.

**Table S3.** Primers used for qPCR to measure the expression level of potato genes.

| **Gene** | **Forward primer sequence** | **Reverse primer sequence** |
| --- | --- | --- |
| *StJAZ* | CAAGTGATTCATCGTCGTCATCGTC | GAAAATCATCAAAAACAATGACTTGTCC |
| *StAOS* | GCTTGTCATAATCTTCTCTTCGCTACTTG | CATTTTCTCCATCGCCGACATCG |
| *StLOX* | GTAGACCACCAACACGAACAGATCC | CAGGGCATGTAATTCAGGGAGG |
| *StERF1*-like | GGATGGTCGCCGTTTAGTTTCAC | CCGCTGTCTAACGCCTCTATAATGTC |
| *StChiA*-like | CAACTACAACTATGGGCCATGTGG | GTTTGTGATGACACCGAATCCAGG |
| *StWin2*-like | GTTCACCTAGCCAAGGTTGCCAG | CGACCACGAGGTCCGACTGG |
| *StEF1a* (reference gene, GenBank: AJ536671.1) | CTTCCTACCTCAAGAAGGTTGGTTAC | CTTGTCTGTTGGTCTCTTGGGC |
| *StAct* (reference gene, GenBank: GQ339765) | GTGAGTCACACTGTCCCTATCTATG | CAGTGGTGGTGAACATATAACCTCT |
